# The Influence of Filament Orientation on Tensile Stiffness in 3D Printed Structures—Numerical and Experimental Studies

**DOI:** 10.3390/ma16155391

**Published:** 2023-07-31

**Authors:** Rafał Bartosiak, Filip Kaźmierczyk, Paweł Czapski

**Affiliations:** Department of Strength of Materials, Faculty of Mechanical Engineering, Lodz University of Technology, Stefanowskiego 1/15, 90-537 Lodz, Polandfilip.kazmierczyk@p.lodz.pl (F.K.)

**Keywords:** additive manufacturing, tensile test, Classical Lamination Theory, Finite Element Method, Representative Volume Element

## Abstract

The present study provides a thorough analysis of the influence of filament orientation on the tensile stiffness of 3D-printed structures. This exploration employs a combination of numerical simulations and experimental trials, providing an extensive understanding of additive manufacturing, particularly 3D printing. This process involves layer-by-layer material deposition to produce three-dimensional objects. The examination specifically targets PLA-based 3D printed structures created using Fused Filament Fabrication (FFF) technology and subjects them to rigorous evaluations using a universal tensile testing machine. Additionally, this approach combines Representative Volume Element (RVE) and Classical Lamination Theory (CLT) techniques to extrapolate the mechanical properties of the test material. Although the initial methodology faces challenges in determining the shear modulus with precision, an in-depth investigation results in enhanced accuracy. Furthermore, this study introduces a parametric RVE numerical method, demonstrating its resilience in handling sensitivity to shear modulus. A comparative study of results derived from both the analytical methods and experimental trials involving five series of samples with varied layups reveals that the newly proposed numerical method shows a stronger correlation with the experimental outcomes, delivering a relative error margin of up to 8%.

## 1. Introduction

Additive manufacturing (AM), often equated with 3D printing [1] in non-technical contexts [2,3], is a transformative approach to industrial production that employs a layer-by-layer addition of materials. The process encompasses a wide array of technologies, including but not limited to binder jetting, material extrusion, and powder bed fusion. The term “additive manufacturing” encapsulates methodologies distinct from traditional subtractive or formative manufacturing techniques. The evolution of AM technologies traces back to the latter half of the 20th century, with significant advancements such as Stereolithography (SLA) [4,5] or Selective Laser Sintering (SLS) [5,6,7]. Specifically, in 1989, Lisa and S. Scott Crump pioneered a material extrusion technique commonly known as Fused Deposition Modeling (FDM) [8,9]. Recognizing its commercial potential, they obtained a patent in 1992 [10] and subsequently founded Stratasys. However, as “Fused Deposition Modeling” and its abbreviation are Stratasys trademarks, the term Fused Filament Fabrication (FFF) has become an alternative descriptor for similar processes [11,12].

The orthotropic nature of FFF printed parts has been emphasized by works like those of Bonada et al. [13], Cuan-Urquizo et al. [14] or Patterson et al. [15,16], who offered multiple approaches of predicting elastic properties of FFF 3D printed parts by investigating the influence of infill pattern on the elastic properties of FFF 3D printed parts or mesoscale modeling. Similar simulations, combined with experimental tests such as those by Fernandez et al., offer predictions on the modulus of elasticity in FFF parts [17]. Additionally, recent advancements like those reported by Kim et al. explore the advancements in FFF 3D printed components [18]. These simulations offer a relatively fast estimation of the elastic properties of FFF-printed structures based only on the structures’ shapes. The most detailed simulation was proposed by Zhou et al. [19] that includes all printing parameters, such as printing temperature field, molding chamber temperature, and nozzle temperature, while most researchers tested it experimentally [20].

With its adaptability and cost-effectiveness, additive manufacturing finds applications across various sectors, including education [21], healthcare [22], and food production [23]. It has also drawn considerable interest from the military sector, which recognizes research and development in additive manufacturing as part of its ongoing technological advancements [24,25].

One significant advantage of additive manufacturing is the ability to predefine an object’s internal structure, leading to mass reduction. Design possibilities with FFF, such as those explored by Sala et al., revolve around custom-made lattice structures [26]. Furthermore, the adaptability of structural designs, as highlighted by Tao et al., aids in producing intricate yet stable structures [27]. Lubombo and Huneault’s work highlights the significance of infill patterns in lightweight cellular structures [28]. This adaptability in design optimizes material usage and reduces production costs. Production costs can also be reduced by recycling the plastics used for printing [29,30].

Due to their unique structure, various approaches have been developed to evaluate the strength properties of 3D-printed objects. The Finite Element Method (FEM) effectively analyzes a model’s mechanical endurance, thermal resistance, or chemical resilience. This process involves discretization, dividing the model into smaller elements for individual inspection. While isotropic objects do not require meticulous attention during discretization, 3D-printed objects, particularly those produced with FFF technology, exhibit a pronounced layer-based structure and orthotropic behavior [31]. The Representative Volume Element (RVE) approach, which discretizes and analyzes a small element of the entire model, can determine various mechanical properties and map the results onto the whole model [32].

Alternatively, sub-modeling refines the mesh only at a specific segment of the entire model, reducing the refinement as the distance from the segment increases [33]. In addition to conventional FEA software, dedicated software for additive manufacturing, like Ansys Additive [34], is emerging. Some researchers also consider isotropy in models printed using FFF technology [35,36].

Objects produced through AM undergo extensive mechanical testing. These tests are in high demand due to the rapidly growing market and ongoing material research. Additionally, the inhomogeneous material composition of the manufactured objects calls for careful consideration of their mechanical properties. Depending on specific requirements, a tensile test of plastic-based coupons can follow ASTM D638 [32,37] or ISO 527 [38] standards.

This study investigates the impact of filament orientation in 3D printed structures on their stiffness properties, specifically Young’s modulus, shear modulus, and Poisson’s ratios. The discussion will commence with a description of experimental procedures, including preliminary tests. These initial tests will establish the necessary parameters of the specimen’s manufacturing process and dimensions for use in the final experiments. The experimental section will be supplemented by identifying a suitable numerical procedure to simulate these parameters. Two hybrid methods will be considered that integrate numerical simulations with analytical calculations: the first involves the Representative Volume Element (RVE) and Classical Lamination Theory (CLT), while the second uses a parametrized raster angle in the RVE simulation and computes elastic properties as a weighted average of the properties of individual layers. The novelty of this approach is that it omits the problems with the determination of shear moduli, which are necessary inputs for CLT. Finally, stress-strain curves, averaged Young’s moduli, and failure stresses of experimental measurements of non-standard raster-angle 3D printed samples will be presented and compared with the outcomes of the two numerical methods.

## 2. Experimental Procedures

### 2.1. Manufacturing of the Samples

**Figure 1:**
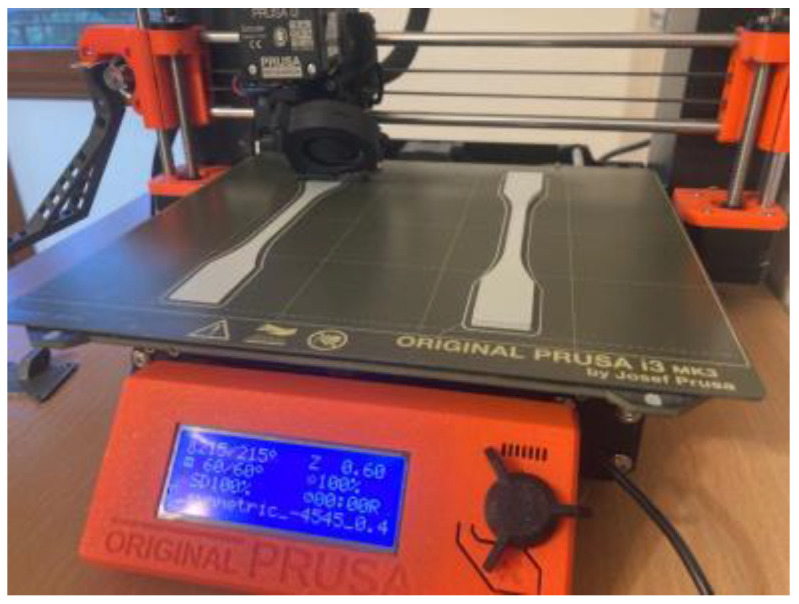
Printing process.

The initial stage encompassed the printing of the samples. The CAD models of the specimens were initially prepared using SolidWorks 2021 academic edition software, in accordance with [39], and later modified to comply with ASTM D638-14 [37]. The G-code was generated using Ultimaker Cura 5.2.2 software, with key parameters highlighted in Table 1. These parameters were not extensively tested prior to the printing process. Models were produced using an Original Prusa i3 MK3S+ printer equipped with a 0.4 mm brass nozzle. The process entailed the simultaneous manufacture of two specimens positioned flat on the printing bed, as depicted in Figure 1. The selected material for this study was Easy PLA Gray, provided by Fiberlogy (Fiberlab S.A.). The material properties of the material according to the technical data sheet delivered by the producer are given in Table 2 [40].

### 2.2. Tensile Testing and Specimen Selection

The printed samples were subsequently categorized and measured before progressing to the second stage of the experimental process. The gauge length section’s thickness and width of each sample were gauged, as these measurements were required as input for the tensile testing machine.

**Figure 2:**
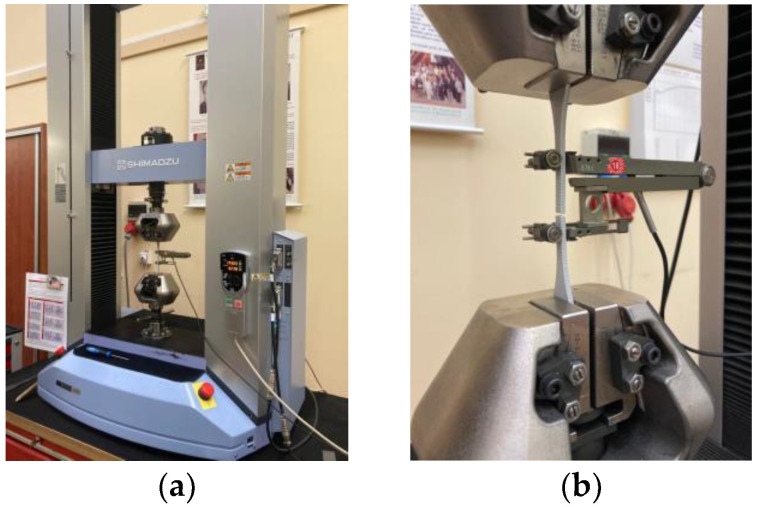
(**a**) View of the testing machine and (**b**) view of the specimen after breaking.

Tensile testing was performed using a Shimadzu AG-X plus table-top type machine with a maximum load capacity of 50 kN, as depicted in Figure 2a. The initiation of measurements involved supplying the thickness and width values of the samples and setting the number of sets and specimens in each set. An extensometer with a 50-mm base distance was employed to ascertain the difference in the specimen’s length before each testing instance. The machine recorded the applied force, including the maximal breaking force, as well as the elongation. A snapshot of a specimen immediately post-testing is illustrated in Figure 2b.

Before reaching the final series of tensile test measurements, three sets of specimens underwent printing and testing. Several factors, such as specimen dimensions and printing parameters, were evaluated during this preliminary investigation.

Initially, the specimen dimensions were presumed to align with those found in reference [40]. A total of six samples were tested, with two assigned to each filament orientation: 0°, 90°, and 90° × 0°. The latter orientation alternates layers consecutively. The specified degrees correspond to the longitudinal direction of force applied to the specimen during testing or, alternatively, to the longer side of the specimen.

Results from this set revealed that the specimens did not fracture within the gauge-length section, indicating the need for a more in-depth analysis.

The second set of tests encompassed a total of 18 specimens. The dimensions were adjusted to match those of Type 1 and Type 2 test specimens per ASTM standards. For each specimen type, nine were tested, with three dedicated to each filament orientation.

**Figure 3:**
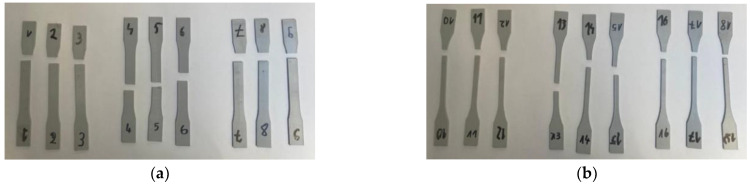
Specimens from the 2nd set of tests: (**a**) Type 1 and (**b**) Type 2.

As seen in the attached Figure 3a,b, only specimens with raster angle 90° (specimen numbers 4, 5, 6 and 13, 14, 15) broke in the gauge length section, whereas incorrect breaking remained for all other filament orientations.

**Figure 4:**
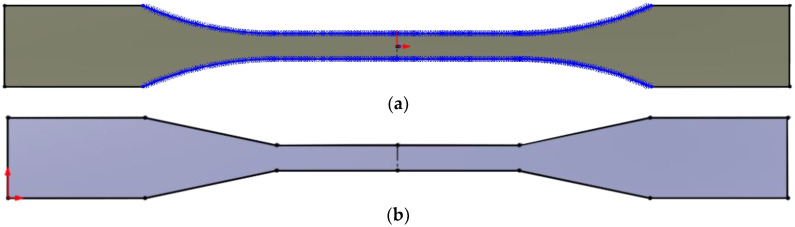
(**a**) Sketch of the specimen Type 2 model with Spline function included, and (**b**) sketch of the specimen Type 2 with “V”-shaped converging lines.

This realization spurred another iteration in the specimen preparation process. For Type 1 specimens, the CAD model was adjusted so that the curves of the specimen’s longer edges were modeled as a single entity, employing the Spline function rather than connecting two arcs and a straight line, as depicted in Figure 4a. Additionally, a new specimen type was tested, which featured “V” shapes in place of the arcs connecting the gauge-length section of the specimen with its wider section, as shown in Figure 4b. Furthermore, during the G-code preparation, the layer width was fine-tuned from 0.4 mm to 0.2 mm. Consequently, the third set consisted of 30 specimens: 18 “V”-shaped specimens with three filament orientations each, varying in layer width (0.2 mm and 0.4 mm), with three per setting; and 12 specimens implementing the Spline function in the CAD model, organized similarly to the “V”-shaped specimens, albeit excluding the 90° orientation since it had already demonstrated proper breakage in the second set of measurements.

**Figure 5:**
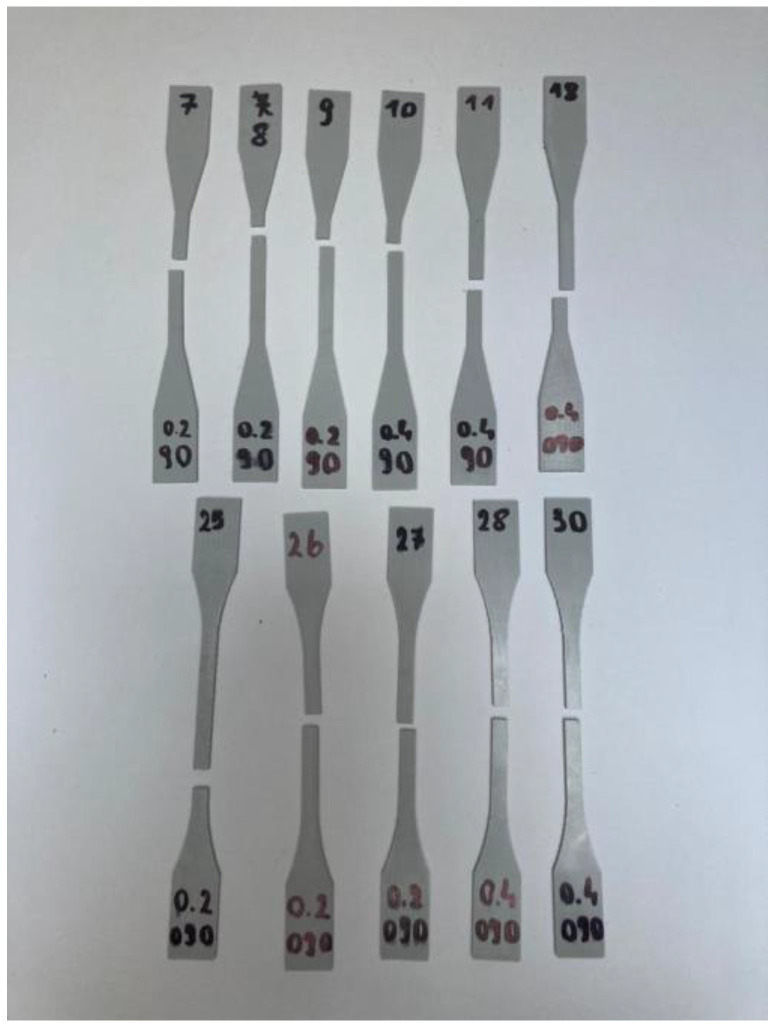
Specimens from the 3rd set of measurements with the correct breaking location.

Despite these alterations, the set of specimens did not yield any significant changes in the breaking location. Most specimens fractured either outside or at the end of the gauge-length section. Figure 5 displays specimens from the third measurement set that broke at the correct location. Their indices are specified at the top of the specimens, with layer width and filament orientation (090 refers to 90° × 0°) notated at the bottom. Therefore, Type 2 specimens in Spline variation have been selected for further testing.

**Figure 6:**
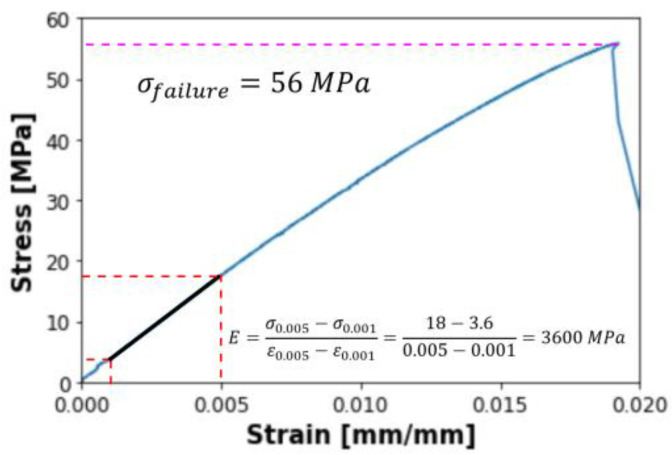
Stress-strain curve for an exemplary sample with a 0° raster angle.

A stress-strain curve was prepared for each sample based on the extensometer results to determine Young’s modulus from sample measurements. Young’s modulus was derived directly from the slope in the linear elastic region. The highest stress value represented the failure load, or the stress at breakage. For instance, Figure 6 illustrates a typical stress-strain curve for a sample with a 0° raster angle. The black line symbolizes Young’s modulus slope, equating to 3600 MPa, calculated as the difference in stress values of 18 and 3.6 and strain values of 0.005 and 0.001. The pink dashed line portrays the failure load, determined from the highest point of the stress-strain curve and transferred to the graph’s vertical axis, resulting in a value of 56 MPa. Similar calculations were made for all other specimens, following the guidelines outlined in the standard [37]. Table 3 summarizes the results from all three sets of preliminary studies.

Calculations were made for Young’s modulus and failure stress for all specimens in each set. Although relatively high standard deviations of the values have been achieved due to various types of tested samples, a strong correlation was observed between these two properties. The values obtained from the calculations align well with the technical data sheet for the filament [16]. The reference provides Young’s modulus and tensile strength at break of 3500 MPa and 53 MPa, respectively, which closely match the values for a 0° filament orientation. This raster angle is closest in strength to isotropic PLA.

### 2.3. Microscopic Testing

Geometry preparation encompassed an examination of the specimen’s microstructure post-breakage. To aid in this investigation, a specimen with a 0° filament orientation was inspected under a microscope.

**Figure 7:**
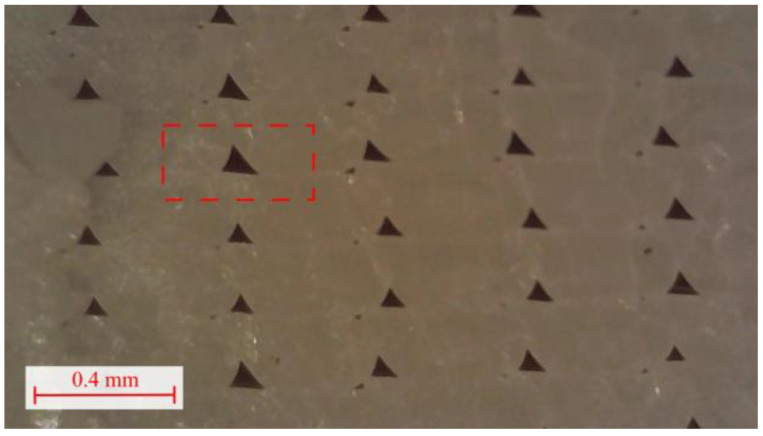
Microstructure of the 3D-printed specimen with 0° filament orientation (red rectangle corresponding to future RVE cell).

The microstructure presented in Figure 7 reveals that the gaps between filament strips are not uniformly homogeneous. The magnitude of these gaps influences the overall strength of a 3D-printed material. Consequently, the largest gap was selected and used as an input for the Representative Volume Element (RVE) model. Figure 7 displays a red square with dashed lines that approximates the cross-sectional area of the RVE model. The overall dimensions of this area align with the layer size, specifically 0.2 mm in height and 0.4 mm in width, in accordance with Figure 7. The exact geometry of the RVE will be discussed more thoroughly in the following sections of this article.

## 3. Numerical and Analytical Studies

This section presents two approaches to predicting the elastic properties (Young’s moduli) of analyzed 3D printed structures with different raster angle layups. The first method combines Representative Volume Element (RVE) and composite materials using Classical Lamination Theory (CLT). In the second approach, RVE modeling is performed across the entire spectrum of raster angles, and the elastic properties of multi-layered 3D prints are found as the weighted average of the properties of individual layers inclined at different angles.

### 3.1. Mixed RVE and CLT Approach

In this approach, based on the microscopic image of the structure, a finite element model of the unit cell is created, and material properties in the main orthotropic directions are determined. Later, composite material CLT is applied to find the elastic properties of multi-layered 3D prints with different raster angle arrangements.

#### 3.1.1. Representative Volume Element Approach

**Figure 8:**
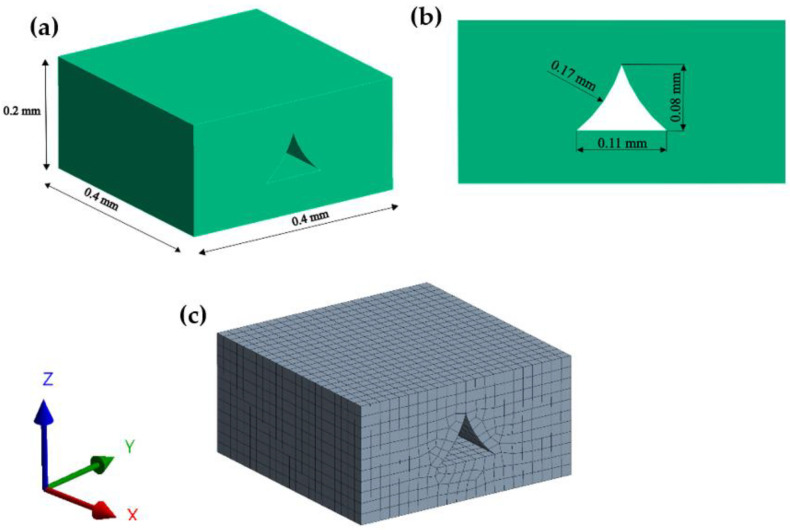
(**a**) Overall dimensions of the model; (**b**) front view; and (**c**) meshed model.

In the initial step, a finite element model of a unit volume of the structure is created based on the microscopic image presented in Figure 7. The model was prepared in SpaceClaim, ANSYS 2022 R1 in-built modeling software, and is presented in Figure 8a,b. As an input material, isotropic PLA properties were used, provided by the manufacturer of the filament [41], specifically: Young modulus E = 3500 MPa, whereas Poisson Ratio ν = 0.35, based on the literature [42,43]. Later, the model was discretized using SOLID186 hexahedral elements [44], with an element size of 0.019 mm, a total number of elements equal to 6615, and 30,681 nodes—see Figure 8c.

**Figure 9:**
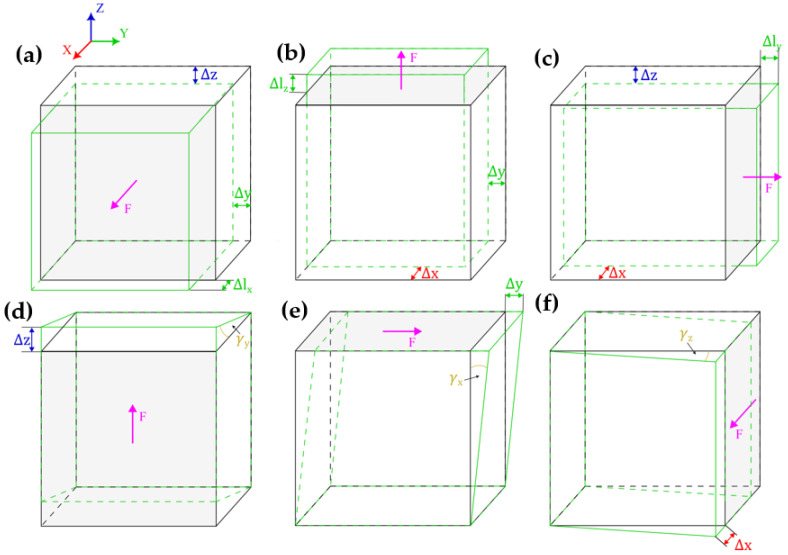
Schematics of 6 simulation variations: tensile are shown in (**a**–**c**), whereas (**d**–**f**) represent shear simulations.

**Figure 10:**
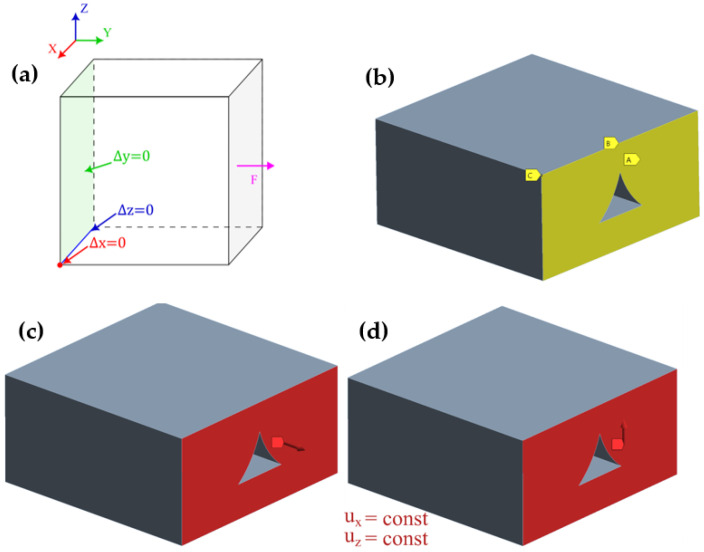
(**a**) Schematics of constraints for all simulations; (**b**) view of constraints extracted from Ansys 2022 R1 software for exemplary tensile or shear simulations; (**c**) view of a force constraint for a tensile simulation; and (**d**) coupling designation applied on the front surface in the case of a shear simulation.

In order to predict all necessary mechanical properties, six simulations with varying load types and directions are performed. The first three simulations were used to determine Young’s moduli in three directions: *E_x_*, *E_y_*, *E_z_*, and Poisson’s ratios *ν_yz_*, *ν_xz_*, and *ν_xy_*—see Figure 9a–c, respectively. The remaining simulations were used to determine shear (Kirchhoff’s) moduli *G_yz_*, *G_xz_*, and *G_xy_*—see Figure 9d–f, respectively. A schematic of the simulation boundary conditions is presented in Figure 10a, supplied with views from Ansys 2022 R1 software of exemplary constraints in tensile and shear simulations in Figure 10b–d.

Tensile simulations were conducted according to the schematic shown in Figure 10a. Three displacement boundary conditions were applied to the RVE model: a surface parallel to the one with force applied; a line and a node, each constraining one degree of freedom, as shown in Figure 10b. When it comes to shear simulations, additional APDL code is needed in order to prevent undesirable deformations. In all three cases, the surface with applied force was coupled in two directions: one parallel and one perpendicular to the direction of the force vector. As shown in Figure 10c,d, force is applied to the surface “front”, whereas u_z_ and u_x_ are directions of node coupling of the surface.

**Figure 11:**
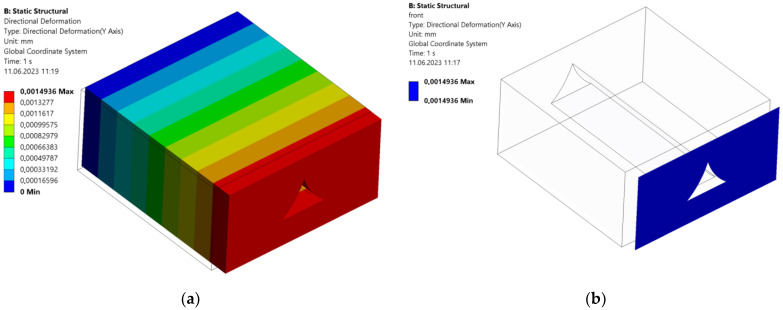
(**a**) Full directional deformation plot of a tensile simulation in the X direction; and (**b**) directional deformation plot only for a front surface; both plots are supplied with respective legends depicting the results of the simulation.

Having prepared and carried out the simulations, results were gathered and used to calculate all nine mechanical properties. Exemplary deformation plots extracted from Ansys software are shown in Figure 11, whereas calculation results are shown in Table 4.

The above results were used as input into an analytical method explained below.

#### 3.1.2. Composites Classical Lamination Theory—Prediction of Young’s Modulus

According to the theory described in [45], Classical Lamination Theory (CLT) is an approach allowing the determination of various mechanical properties of an orthotropic composite material. A laminate is an organized pile of uni-directional, bonded plies (or layers) of composite materials with a given direction of its fibers. In addition, the layers have much bigger lengths than thicknesses. Since orthotropic material is considered, the direction of stiffness, i.e., Young’s moduli, needs to be taken into account:

(1) $$E_{1}=\frac{\sigma_{1}}{\varepsilon_{1}}\;\mathrm{and}\;E_{2}=\frac{\sigma_{2}}{\varepsilon_{2}}$$

where 1 and 2 subscripts refer to the direction of a property in relation to the direction of the plies, parallel and perpendicular, respectively. Analogically, shear forces are defined using shear (Kirchhoff’s) modulus, which links shear stress τ with shear strain γ:

(2) $$\tau_{12}=\gamma_{12}G_{12}$$

In the case of plane stress, namely, a plate undergoing stresses in various directions within a plane, Poisson’s ratios need to be considered:

(3) $$\nu_{12}=\frac{\varepsilon_{2}}{\varepsilon_{1}}\;\mathrm{and}\;\nu_{21}=\frac{\varepsilon_{1}}{\varepsilon_{2}}$$

Using all the properties mentioned above, a matrix equation can be derived:

(4) $$\left[\begin{array}{c} \sigma_{1}\\ \sigma_{2}\\ \tau_{12} \end{array}\right]=\left[\begin{array}{ccc} Q_{11} & Q_{12} & 0\\ Q_{12} & Q_{22} & 0\\ 0 & 0 & Q_{66} \end{array}\right]\left[\begin{array}{c} \varepsilon_{1}\\ \varepsilon_{2}\\ \gamma_{12} \end{array}\right]$$

The 3 × 3 matrix, called the compliance matrix, consists of various *Q*’s, which are reduced stiffnesses:

(5) $$Q_{11}=\frac{E_{1}}{1-\nu_{12}\nu_{21}}$$

(6) $$Q_{22}=\frac{E_{2}}{1-\nu_{12}\nu_{21}}$$

(7) $$Q_{12}=\frac{\nu_{21}E_{1}}{1-\nu_{12}\nu_{21}}=\frac{\nu_{12}E_{2}}{1-\nu_{12}\nu_{21}}$$

(8) $$Q_{66}=G_{12}$$

To transform the stresses and strains into the principal material directions, the matrix takes the form:

(9) $$\left[\begin{array}{c} \sigma_{x}\\ \sigma_{y}\\ \tau_{xy} \end{array}\right]=\left[\begin{array}{ccc} \overset{̿}{Q_{11}} & \overset{̿}{Q_{12}} & \overset{̿}{Q_{16}}\\ \overset{̿}{Q_{12}} & \overset{̿}{Q_{22}} & \overset{̿}{Q_{26}}\\ \overset{̿}{Q_{16}} & \overset{̿}{Q_{26}} & \overset{̿}{Q_{66}} \end{array}\right]\left[\begin{array}{c} \varepsilon_{x}\\ \varepsilon_{y}\\ \gamma_{xy} \end{array}\right]$$

where *x* and *y* are axes corresponding to the direction of the applied load. Components in the lamina stress matrix for a ply with fibers oriented at θ may be represented as:

(10) $$\bar{Q_{11}}=Q_{11}m^{4}+2\left(Q_{12}+2Q_{66}\right)m^{2}n^{2}+Q_{22}n^{4}$$

(11) $$\bar{Q_{12}}=\left(Q_{11}+Q_{22}-4Q_{66}\right)m^{2}n^{2}+Q_{12}\left(m^{4}+n^{4}\right)$$

(12) $$\bar{Q_{22}}=Q_{11}n^{4}+2\left(Q_{12}+2Q_{66}\right)m^{2}n^{2}+Q_{22}m^{4}$$

(13) $$\bar{Q_{16}}=\left(Q_{11}-Q_{12}-2Q_{66}\right)m^{3}n+\left(Q_{12}-Q_{22}+2Q_{66}\right)mn^{3}$$

(14) $$\bar{Q_{26}}=\left(Q_{11}-Q_{12}-2Q_{66}\right)n^{3}m+\left(Q_{12}-Q_{22}+2Q_{66}\right)nm^{3}$$

(15) $$\bar{Q_{66}}=\left(Q_{11}+Q_{22}-2Q_{12}-2Q_{66}\right)m^{2}n^{2}+Q_{66}(m^{4}+n^{4})$$

where m=cos⁡θ and n=sin⁡θ.

It can be seen that the resultant stress will produce both normal and shear stress on the fibers of the laminate, even in the case of a normal load applied to the whole laminate.

Matrices denoted as *A*, *B*, and *D* are introduced to arrange all possible loads acting on the laminate. They correspond to the thickness of the ply, its distance from the midplane of the laminate, and specific loads. Hence, the A matrix, namely, the extensional stiffness matrix, relates to normal stresses and strains. Taking into account the geometry of the layers and the whole laminate in this research, the second term of the matrix shall be represented as $t_{k}$:

(16) $$A_{ij}=\sum_{k=1}^{n}{\left[{\bar{Q}}_{ij}\right]}_{k}\left(h_{k}-h_{k-1}\right)$$

(17) $$A_{ij}=\sum_{k=1}^{n}{\left[{\bar{Q}}_{ij}\right]}_{k}t_{k}$$

Similarly, the coupling stiffness matrix, namely the *B* matrix, relates bending strains to normal stress, or vice versa:

(18) $$B_{ij}=\frac{1}{2}\sum_{k=1}^{n}{\left[{\bar{Q}}_{ij}\right]}_{k}\left(h_{k}^{2}-h_{k-1}^{2}\right)$$

(19) $$B_{ij}=\sum_{k=1}^{n}{\left[{\bar{Q}}_{ij}\right]}_{k}t_{k}\frac{h_{k}+h_{k-1}}{2}\;$$

Eventually, the *D* matrix, which is called the bending stiffness matrix, corresponds to the number of plate curvatures and the bending moments:

(20) $$D_{ij}=\frac{1}{3}\sum_{k=1}^{n}{\left[{\bar{Q}}_{ij}\right]}_{k}\left(h_{k}^{3}-h_{k-1}^{3}\right)$$

(21) $$D_{ij}=\sum_{k=1}^{n}{\left[{\bar{Q}}_{ij}\right]}_{k}\left(\frac{t_{k}^{3}}{12}+t_{k}\bar{z_{k}^{2}}\right)\;$$

Young’s Modulus $E_{x}$, can be determined using components from all three matrices mentioned above and strain in the *x* direction:

(22) $$\frac{N_{x}lh}{\varepsilon_{x}^{0}}=E_{x}=\frac{1}{h}\frac{\left|\begin{array}{cccccc} A_{11} & A_{12} & A_{16} & B_{11} & B_{12} & B_{16}\\ A_{12} & A_{22} & A_{26} & B_{12} & B_{22} & B_{26}\\ A_{16} & A_{26} & A_{66} & B_{16} & B_{26} & B_{66}\\ B_{11} & B_{12} & B_{16} & D_{11} & D_{12} & D_{16}\\ B_{12} & B_{22} & B_{26} & D_{12} & D_{22} & D_{26}\\ B_{16} & B_{26} & B_{66} & D_{16} & D_{26} & D_{66} \end{array}\right|}{\left|\begin{array}{ccccc} A_{22} & A_{26} & B_{12} & B_{22} & B_{26}\\ A_{26} & A_{66} & B_{16} & B_{26} & B_{66}\\ B_{12} & B_{16} & D_{11} & D_{12} & D_{16}\\ B_{22} & B_{26} & D_{12} & D_{22} & D_{26}\\ B_{26} & B_{66} & D_{16} & D_{26} & D_{66} \end{array}\right|}$$

Young’s Modulus in the y direction $E_{y}$, and shear modulus $G_{xy}$, can be found similarly with the use of terms from the *A*, *B*, and *D* matrices:

(23) $$\frac{N_{y}lh}{\varepsilon_{y}^{0}}=E_{y}=\frac{1}{h}\frac{\left|\begin{array}{cccccc} A_{11} & A_{12} & A_{16} & B_{11} & B_{12} & B_{16}\\ A_{12} & A_{22} & A_{26} & B_{12} & B_{22} & B_{26}\\ A_{16} & A_{26} & A_{66} & B_{16} & B_{26} & B_{66}\\ B_{11} & B_{12} & B_{16} & D_{11} & D_{12} & D_{16}\\ B_{12} & B_{22} & B_{26} & D_{12} & D_{22} & D_{26}\\ B_{16} & B_{26} & B_{66} & D_{16} & D_{26} & D_{66} \end{array}\right|}{\left|\begin{array}{ccccc} A_{11} & A_{16} & B_{11} & B_{12} & B_{16}\\ A_{16} & A_{66} & B_{16} & B_{26} & B_{66}\\ B_{11} & B_{16} & D_{11} & D_{12} & D_{16}\\ B_{12} & B_{26} & D_{12} & D_{22} & D_{26}\\ B_{16} & B_{66} & D_{16} & D_{26} & D_{66} \end{array}\right|}$$

(24) $$\frac{N_{xy}lh}{\gamma_{xy}^{0}}=G_{xy}=\frac{1}{h}\frac{\left|\begin{array}{cccccc} A_{11} & A_{12} & A_{16} & B_{11} & B_{12} & B_{16}\\ A_{12} & A_{22} & A_{26} & B_{12} & B_{22} & B_{26}\\ A_{16} & A_{26} & A_{66} & B_{16} & B_{26} & B_{66}\\ B_{11} & B_{12} & B_{16} & D_{11} & D_{12} & D_{16}\\ B_{12} & B_{22} & B_{26} & D_{12} & D_{22} & D_{26}\\ B_{16} & B_{26} & B_{66} & D_{16} & D_{26} & D_{66} \end{array}\right|}{\left|\begin{array}{ccccc} A_{11} & A_{12} & B_{11} & B_{12} & B_{16}\\ A_{12} & A_{22} & B_{12} & B_{22} & B_{26}\\ B_{11} & B_{12} & D_{11} & D_{12} & D_{16}\\ B_{12} & B_{22} & D_{12} & D_{22} & D_{26}\\ B_{16} & B_{26} & D_{16} & D_{26} & D_{66} \end{array}\right|}$$

In order to derive an equation for Poisson’s Ratios, strain in the *y* direction while stress is applied in the *x* direction is needed:

(25) $$\varepsilon_{y}^{0}=-N_{x}\frac{\left|\begin{array}{ccccc} A_{12} & A_{26} & B_{12} & B_{22} & B_{26}\\ A_{16} & A_{66} & B_{16} & B_{26} & B_{66}\\ B_{11} & B_{16} & D_{11} & D_{12} & D_{16}\\ B_{12} & B_{26} & D_{12} & D_{22} & D_{26}\\ B_{16} & B_{66} & D_{16} & D_{26} & D_{66} \end{array}\right|}{\left|\begin{array}{cccccc} A_{11} & A_{12} & A_{16} & B_{11} & B_{12} & B_{16}\\ A_{12} & A_{22} & A_{26} & B_{12} & B_{22} & B_{26}\\ A_{16} & A_{26} & A_{66} & B_{16} & B_{26} & B_{66}\\ B_{11} & B_{12} & B_{16} & D_{11} & D_{12} & D_{16}\\ B_{12} & B_{22} & B_{26} & D_{12} & D_{22} & D_{26}\\ B_{16} & B_{26} & B_{66} & D_{16} & D_{26} & D_{66} \end{array}\right|}$$

Then, $\nu_{xy}$ will be expressed as a ratio of strains in the *x* and *y* directions, respectively:

(26) $$\nu_{yx}=-\frac{\varepsilon_{x}^{0}}{\varepsilon_{y}^{0}}=-\frac{\left|\begin{array}{ccccc} A_{12} & A_{16} & B_{11} & B_{12} & B_{16}\\ A_{16} & A_{66} & B_{16} & B_{26} & B_{66}\\ B_{12} & B_{16} & D_{11} & D_{12} & D_{16}\\ B_{22} & B_{26} & D_{12} & D_{22} & D_{26}\\ B_{16} & B_{66} & D_{16} & D_{26} & D_{66} \end{array}\right|}{\left|\begin{array}{ccccc} A_{11} & A_{16} & B_{12} & B_{22} & B_{26}\\ A_{16} & A_{66} & B_{16} & B_{26} & B_{66}\\ B_{11} & B_{16} & D_{11} & D_{12} & D_{16}\\ B_{12} & B_{26} & D_{12} & D_{22} & D_{26}\\ B_{16} & B_{66} & D_{16} & D_{26} & D_{66} \end{array}\right|}$$

The equations presented above were used to determine all necessary mechanical properties using data obtained from the numerical simulations of the RVE model.

### 3.2. Angular RVE—Raster Angle Parametrized Model

A second numerical approach was also proposed to supplement the methods mentioned earlier. Based on the geometry presented in Section 3.1.1, a parameterized simulation was prepared to determine the behavior of the RVE’s mechanical properties across the entire spectrum of raster angles.

**Figure 12:**
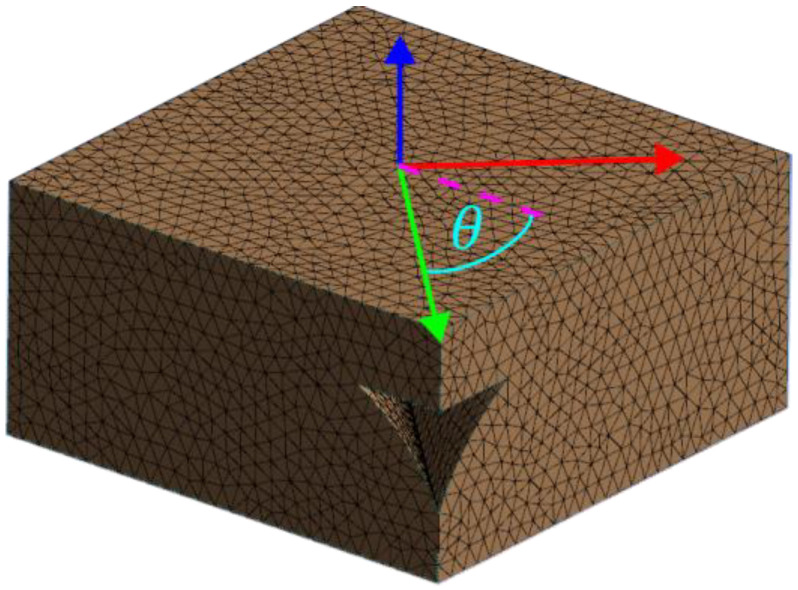
Mesh of an angular RVE model.

The geometry remained unchanged; nevertheless, the mesh was prepared again. A SOLID187 tetrahedral element with midside nodes was used in this case [44], achieving 90,194 elements and 134,355 nodes. The mesh is depicted in Figure 12; the case shown is for a force applied at an angle θ = 45°. 

**Figure 13:**
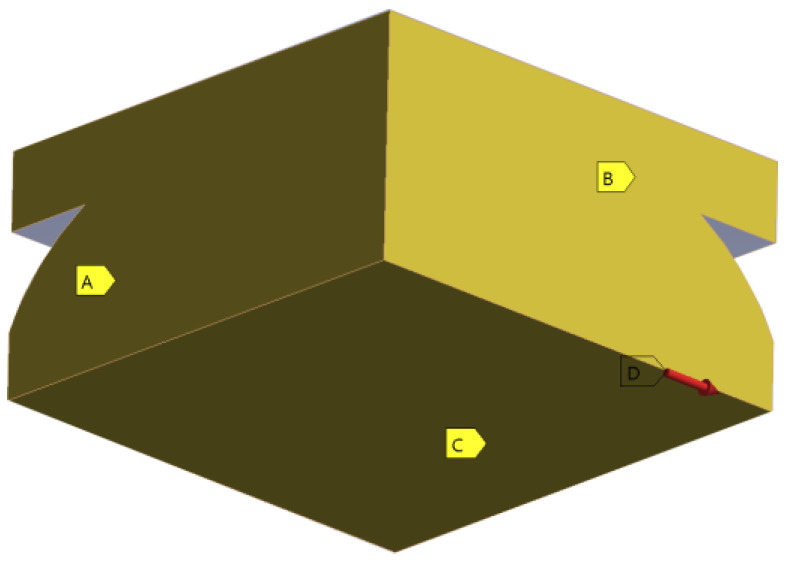
Boundary conditions of the angular RVE model.

The simulation itself was conducted similarly to a regular RVE. Restraining three surfaces in their normal directions, shown in Figure 13 as yellow surfaces from displacement, was sufficient for boundary conditions.

The parameter in the simulation was set directly in the model’s geometry. It is based on rotation around a vertical axis, as seen in Figure 12. The step size was set to 2.5°, which returned 36 × 6 = 216 simulations. During the simulations, necessary data was preserved, namely: three surface areas (relating to the front, top, and side walls of the model) and displacements; in tensile simulations, one per each axis, nine in total; in shear simulations, one per each simulation, three in total.

All this data was necessary for calculating the averaged Young’s modulus, carried out in the second step. In order to do that, one has to familiarize with the methodology explained below.

The specimens tested in this study consist of 16 layers of nearly homogenous filament strips. Initially, one filament layer will be considered. From Hooke’s Law:

(27) $$∆l=\frac{Fl}{Ewt}$$

where *F* is the tensile force acting on the layer; *l* is the initial length of the layer; *w* is the width of the layer; *t* is the thickness of the layer; and ∆l is the displacement of the layer.

Solving for the force in Equation (27) produces:

(28) $$F=\frac{Ewt∆l}{l}$$

One can translate this equation to several layers, representing it as a sum of forces per layer, even though the force magnitude at a specific layer is unknown.

(29) $$F_{1}+F_{2}+...+F_{n}=\sum_{i=1}^{n}F_{i}$$

where *n* is the number of layers.

Implementing Equation (28) into Equation (29), while bearing in mind that all components, in addition to Young’s modulus and the layer thickness, of Equation (28) are constant for all layers, Equation (29) can be represented as a sum of Young’s moduli multiplied by consecutive thicknesses:

(30) $$E_{1}t_{1}+E_{2}t_{2}+...E_{n}t_{n}=\sum_{i=1}^{n}E_{i}t_{i}$$

**Figure 14:**
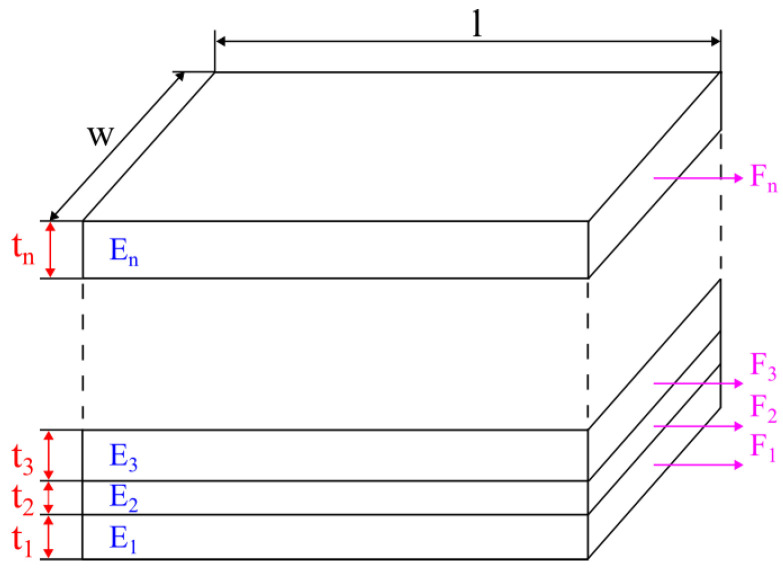
Schematic of the calculation method used for angular RVE.

The technique is supplied with a schematic shown in Figure 14. Using such an approach, both orthotropic Young’s moduli $E_{1}$ and $E_{2}$, were calculated based on the data obtained from simulations of angular RVE.

### 3.3. Comparison of the Models

**Figure 15:**
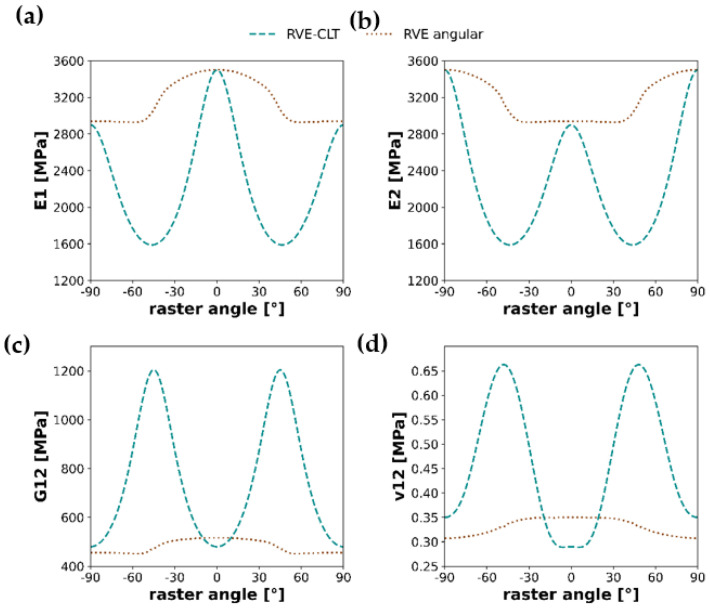
Graphs of orthotropic material properties: (**a**) Young’s modulus $E_{1}$, (**b**) Young’s modulus $E_{2}$, (**c**) Shear modulus, and (**d**) Poisson’s ratio $\nu_{12}$; shown as a comparison between the RVE-CLT approach and angular RVE methodology.

In this section, calculations results for the whole spectrum of raster angles of the two methods, RVE-CLT and angular RVE, will be compared based on plots from Figure 15.

The above graphs show the results of Young’s moduli, shear modulus, and Poisson ratio calculated for the two methods presented earlier in the study. Both methods, when analyzed separately, preserve a specific behavior; for instance, in the case of RVE-CLT, for both Young moduli $E_{1}$ and $E_{2}$, lowest raster angles are for 45° and −45°, whereas in the vicinity of the same raster angles, plots of angular RVE depict either an increase or decrease. Nevertheless, more detailed conclusions cannot be drawn from either of the four pairs of plots, as they do not depict any similar behavior.

### 3.4. RVE-CLT Approach—Troubleshooting

As shown in Figure 15, it is visible that the mixed RVE-CLT approach significantly deviates from values of Young’s moduli in the case of samples with layups different than [0,0] and [90,90]. Troubleshooting of this issue will be performed based on the workflow analysis of this approach. First, it is worth noticing that the input parameters for the RVE finite element model are isotropic, elastic properties of the material (Young’s modulus *E* and Poisson’s ratio *ν*). The output of RVE modeling used as an input to Classical Lamination Theory are Young’s moduli in two orthotropic directions, *E*_1_ and *E*_2_, shear modulus *G*_12_, and Poisson’s ratio *ν*_12_. Bearing in mind the results of the preliminary study, it is worth noting that the result of *E*_2_, obtained from RVE (*E*_2,*RVE*_ = 2938 MPa), can be treated as expected. It means that potential failure in the approach can be hidden in the determination of shear modulus *G*_12_ or Poisson’s ratio *ν*_12_. On the other hand, Poisson’s ratio is a relatively simple quantity to determine as a fraction of lateral shortening to elongation of the sample. Hence, it suggests that a solution should be sought to determine the shear modulus.

(31) $$G=\frac{\tau }{\gamma }=\frac{\frac{F}{A}}{\frac{∆x}{l}}=\frac{Fl}{A∆x}$$

**Figure 16:**
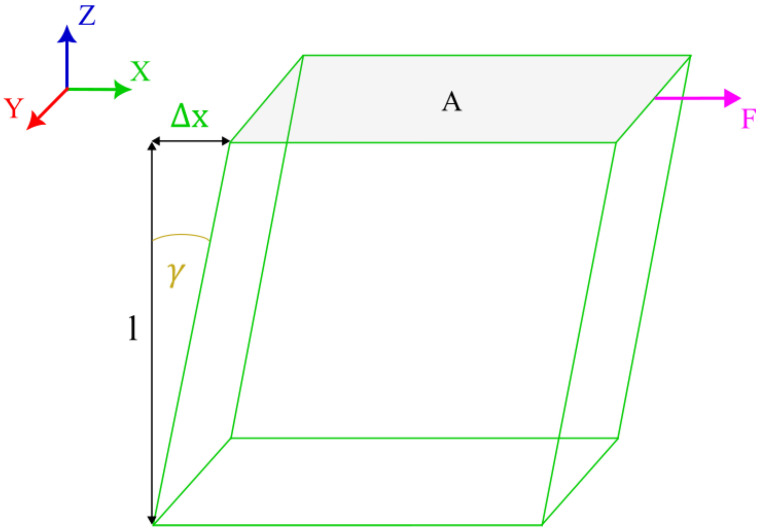
Schematic of a cube under shear.

Equation (31) supplied with a schematic from Figure 16 defines the shear modulus as a ratio of shear stress and strain. On the other hand, for isotropic materials, the relationship between Young’s modulus, Poisson’s ratio, and shear modulus is given as [46]:

(32) $$G=\frac{E}{2(1+\nu )}$$

**Figure 17:**
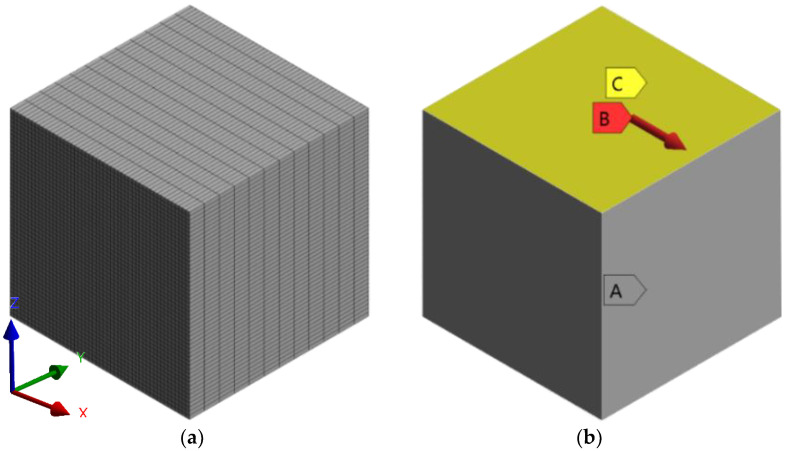
(**a**) Meshed RVE steel cube and (**b**) boundary conditions of the cube.

To see if the numerical model is constructed correctly, the RVE model will be prepared for a well-known material—structural steel. A FE model of shearing (shear force *F* = 100 N) of the cube of 10 mm × 10 mm × 10 mm made from steel with *E* = 200 GPa and *ν* = 0.3 is prepared and presented in Figure 17a,b.

Based on this model, the shear modulus is equal to:

(33) $$G=\frac{\tau }{\gamma }=\frac{Fl}{A∆x}=\frac{100\;N\cdot 10\;mm}{100\;{mm}^{2}\cdot 0.00019158\;mm}=52.19\;GPa$$

On the other hand, according to Equation (32), the shear modulus for steel is equal to:

(34) $$G_{steel}=\frac{E}{2(1+\nu )}=\frac{200\;GPa}{2(1+0.3)}=76.92\;GPa\;$$

**Figure 18:**
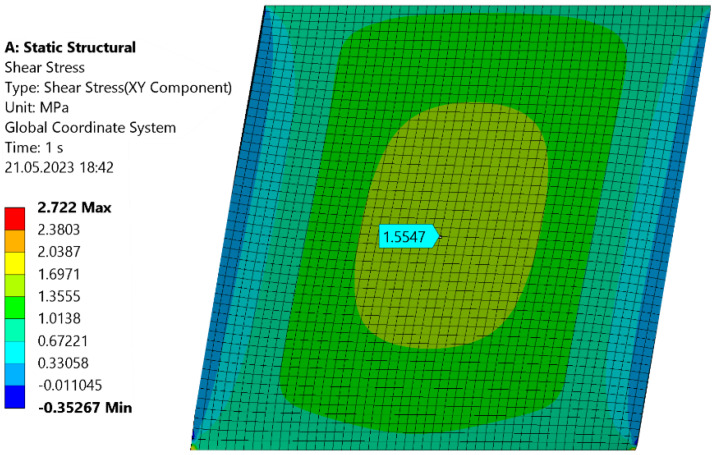
In-plane shear resultant stress of a steel cube.

This discrepancy indicates that the numerical model must be carefully reviewed. To do it, in-plane shear stress is displayed:

Figure 18 shows that the shear stress in the middle of the wall is different than on its boundaries, which can be explained by Saint-Venant’s principle [46]. Taking the value of shear stress from the middle of the wall and substituting it with the formula for shear modulus:

(35) $$G=\frac{\tau }{\gamma }=\frac{\tau }{\frac{∆x}{l}}=\frac{1.5547\;MPa}{\frac{0.00019158\;mm}{10\;mm}}=81.15\;GPa$$

is an acceptable result. Applying this observation to the 3D printed structures analyzed in this study, one obtains:

(36) $$G_{PLA,FEM}=\frac{\tau }{\gamma }=\frac{\tau }{\frac{∆x}{l}}=817\;MPa$$

Since the RVE model is assumed to be isotropic, it is possible to use the analytical formula:

(37) $$G_{PLA,theory}=\frac{E}{2(1+\nu )}=\frac{3500\;MPa}{2(1+0.35)}=1296\;MPa\;$$

Substituting these results to the Classical Lamination Theory and confronting them with the angular RVE approach, one obtains updated graphs of orthotropic material properties.

**Figure 19:**
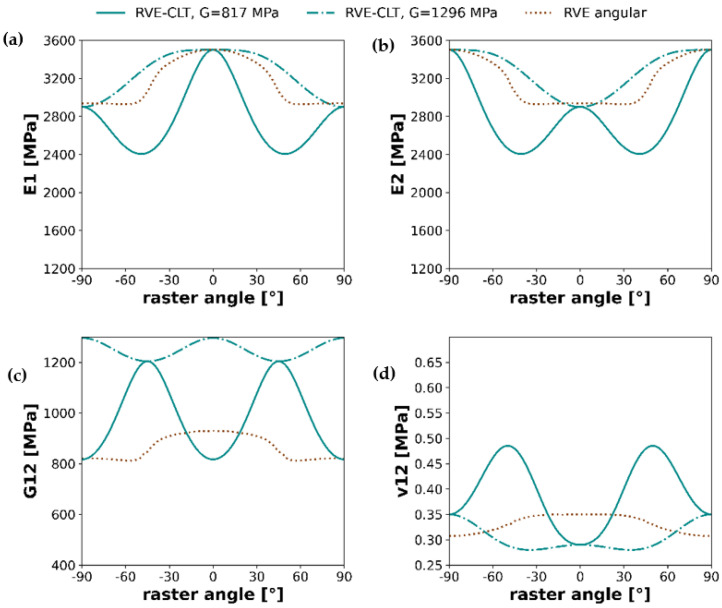
Graphs of orthotropic material properties: (**a**) Young’s modulus $E_{1}$, (**b**) Young’s modulus $E_{2}$, (**c**) Shear modulus, and (**d**) Poisson’s ratio $\nu_{12}$; shown as a comparison between the angular RVE approach and two variants of RVE-CLT approaches.

Similarly to Figure 15, the graphs shown in Figure 19 represent the results of Young’s moduli, shear modulus, and Poisson ratio calculated, however, with an altered value of shear modulus. In this case, the RVE angular and RVE-CLT plots correspond much better. Full-spectrum plots of Young’s moduli maintain similar behavior, with either increase or decrease in the vicinity of 45° or −45° raster angles, whereas interchanging peaks at 90°, −90° and 0, for instance $E_{1}$ attains its maximum value at 0°, on the other hand, at 0°, $E_{2}$ attains its minimum. Shear modulus and Poisson’s ratio plots correspond slightly worse, yet after changing the methodology for shear modulus determination, the situation is improved, especially in the case of Poisson’s ratio.

## 4. Results

In this section, the results of experimental studies will be presented, including stress-strain curves, averaged Young’s moduli, and failure stresses. In the final stage, stiffnesses will be confronted with predictions obtained from an adjusted RVE-CLT methodology as well as the RVE method with a raster angle parametrized model (angular RVE).

### 4.1. Experimental Results

Bearing in mind the preliminary tests, a set containing 50 specimens was 3D printed, preserving all machine settings, dimensions, CAD models, and printing process settings. Since the layer height was set to 0.2 mm and the specimens were 3.2 mm thick, they consisted of 16 layers. Five series, 10 specimens each, of different layups were examined:

- Series 1—layup of [0,0]_8_, denoted as S1 [0,0];
- Series 2—layup of [90,90]_8_, denoted as S2 [90,90];
- Series 3—layup of [30,−30]_4s_, denoted as S3 [30,−30];
- Series 4—layup of [45,−45]_4s_, denoted as S4 [45,−45];
- Series 5—layup of [60,−60]_4s_, denoted as S6 [60,−60].

It means that in the case of the first two layups, the layers maintained the same raster angle for all 16 layers. Series S3, S4, and S5 included interchanging layers with a symmetry plane between the 8th and 9th layers, bearing in mind that the raster angle is measured according to the symmetry axis of the specimen, or, in other words, the longer side of the sample.

**Figure 20:**
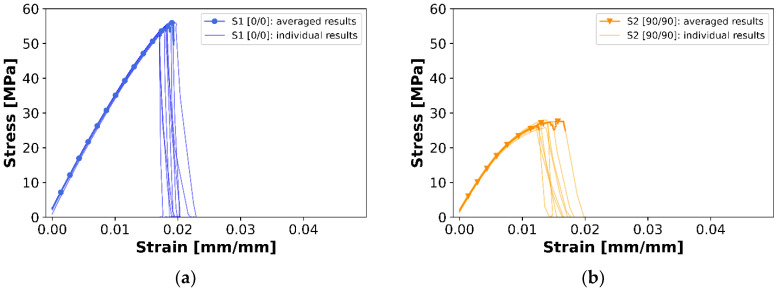

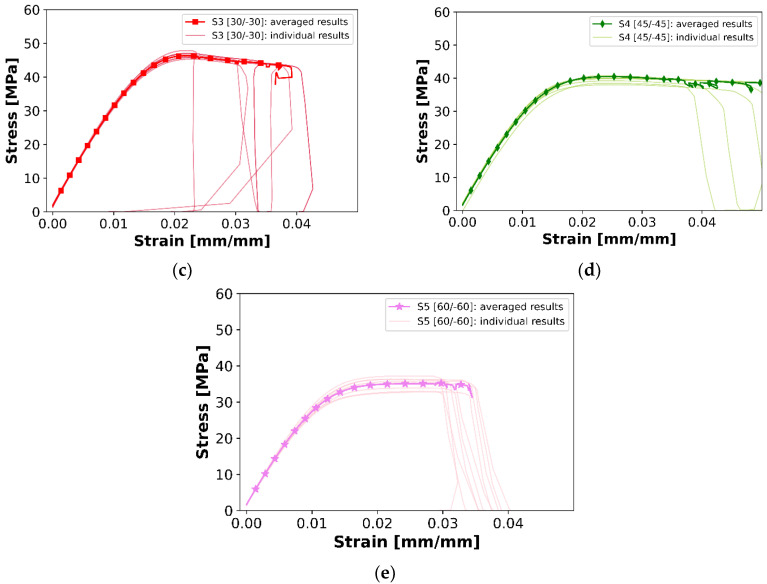
Individual and averaged experimental curves (calculated as rolling average) for (**a**) S1([0,0]) (**b**) S2([90,90]), (**c**) S3([30,−30]), (**d**) S4([−45,45]), and (**e**) S5([60,−60]) layups.

**Figure 21:**
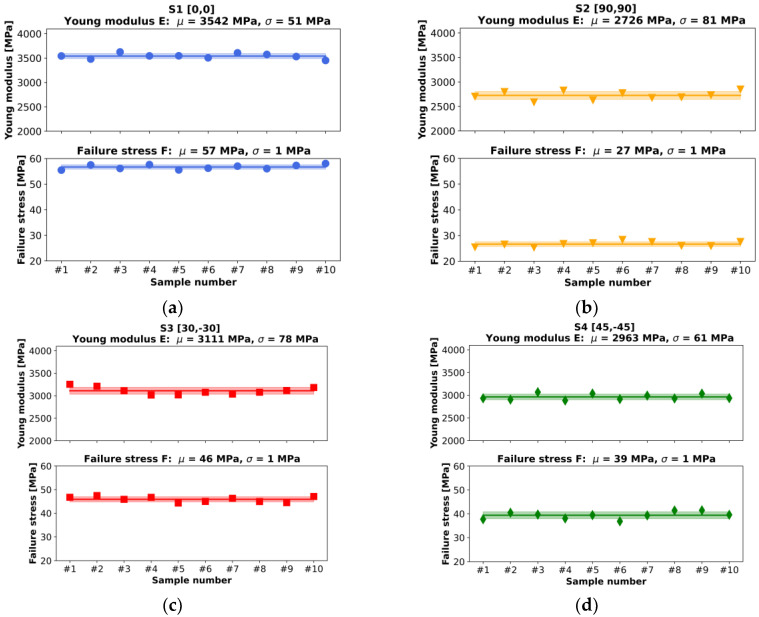

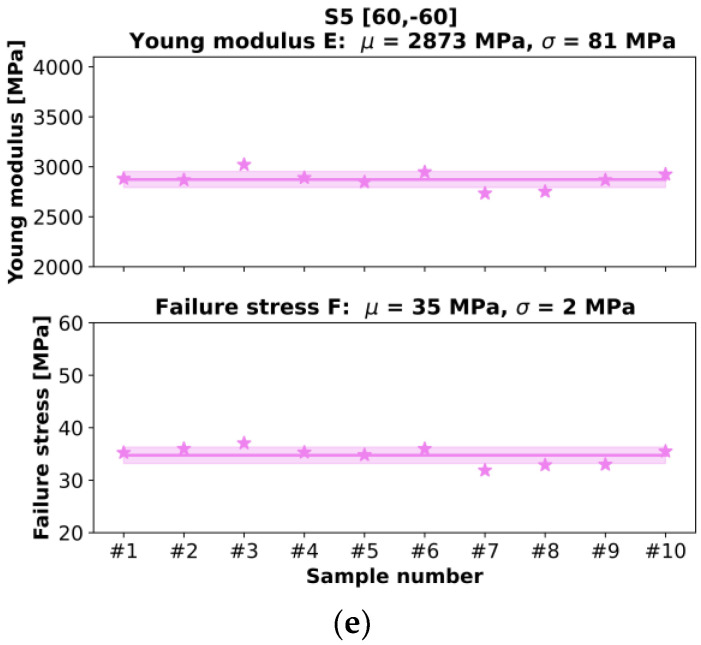
Average Young’s moduli and failure stresses with corresponding standard deviations for (**a**) S1([0,0]) (**b**) S2([90,90]), (**c**) S3([30,−30]), (**d**) S4([−45,45]), and (**e**) S5([60,−60]) layups.

**Figure 22:**
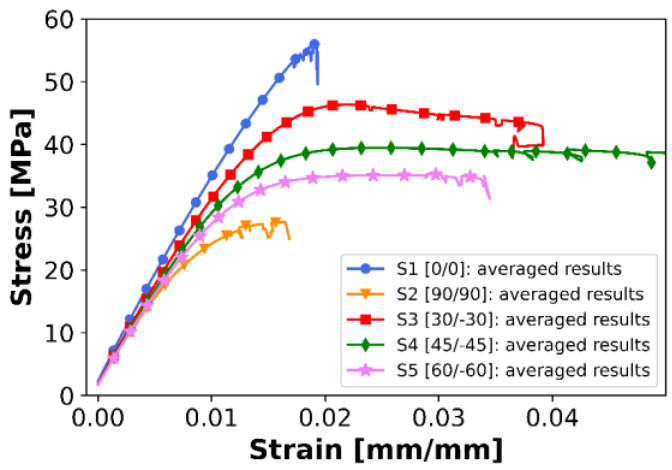
Comparison of averaged tensile curves for all analyzed layups (calculated as a rolling average from all experimental curves in each series of samples).

**Figure 23:**
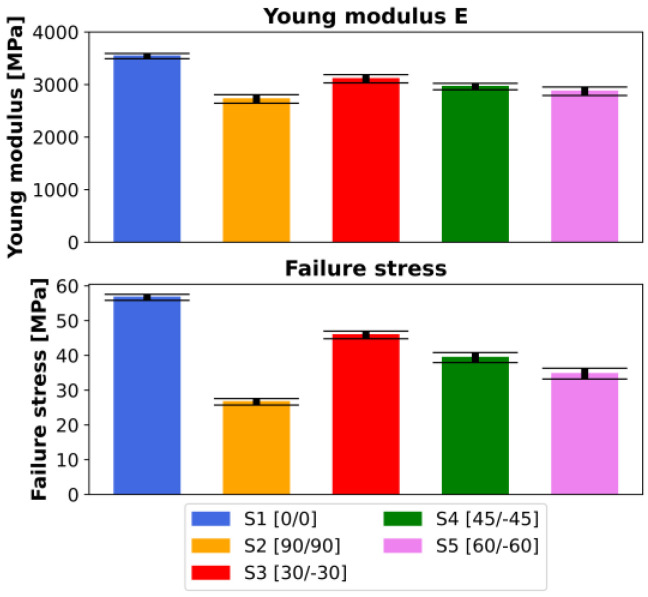
Comparison of averaged Young’s moduli and failure stresses for all analyzed layups with corresponding standard deviations.

Figure 20, Figure 21, Figure 22 and Figure 23 present the results of the experimental procedures performed in this study. The plots in Figure 20 and Figure 22 illustrate stress and strain relationships for all samples. These relationships can be discerned by analyzing the plots for specific layups. The highest stress before failure was observed in the [0/0] layup, while the lowest was noted for [90/90]. Conversely, other layups displayed significantly higher strains at failure, ranging from 0.03 to 0.04, compared to a maximum value of 0.02 in S1. This could be attributed to the interchanging layers of these samples. One plot that significantly deviates from the others in terms of strain is the [−45/45] layup. For most samples, the strain exceeded 0.04, which is markedly higher than other layups. Normal and shear forces in this particular arrangement led to the largest plastic deformation. A notable distinction between S1 and S2, compared to the other series, is the uniformity in the samples’ behavior under tensile stress. Plots of individual results, indicated by thin lines on the graphs, demonstrate that failure occurred over a broader range of strains in the S3, S4, and S5 sets compared to the first two.

When comparing all average results of stress and strain relationships, as displayed in Figure 22, a nearly pure brittle fracture was observed in the case of 0° filament orientation. However, increased brittleness was impacted by the de-bonding of the filament strips in a sample with a [90/90] layup, as the force direction was perpendicular to the orientation of the filament strips.

Average Young’s moduli and failure stresses are documented in Figure 21 and Figure 23. The highest Young’s modulus was obtained for S1, equating to 3542 ± 51 MPa, with a decrease observed for increasing filament angles, reaching 2726 ± 81 MPa in the [90,90] layup. This also correlates to the stress vs. strain plots. The distribution of Young’s moduli magnitudes relates to the highest measured stresses. Although the angular difference between the 0° and 30° filament orientations is the same as for the last two pairs, the difference in Young’s moduli is much higher for the first pair, approximately 300 MPa compared to 150 MPa. This could be due to the type of stress experienced in the [0,0] filament orientation. A specimen subjected to pure axial stress, where shear stress is absent, results in a significantly higher strength than any other orientation. In addition to pure tensile (0°) and shear (90°) loads, the laminate is subjected to both. Given that interlayer bonding in the 90° orientation needs to be taken into account, the overall strength will be considerably lower.

In all instances, failure stress corresponds to the obtained Young’s modulus, with insignificant or very low standard deviations. Again, as per the graphs, specimens with a 0° orientation failed under the highest stress, amounting to 57 ± 1 MPa, while 90° specimens failed the quickest—under only 27 ± 1 MPa. A range of fracture types were observed in the study.

### 4.2. Experimental vs. Numerical Results—Comparison

**Figure 24:**
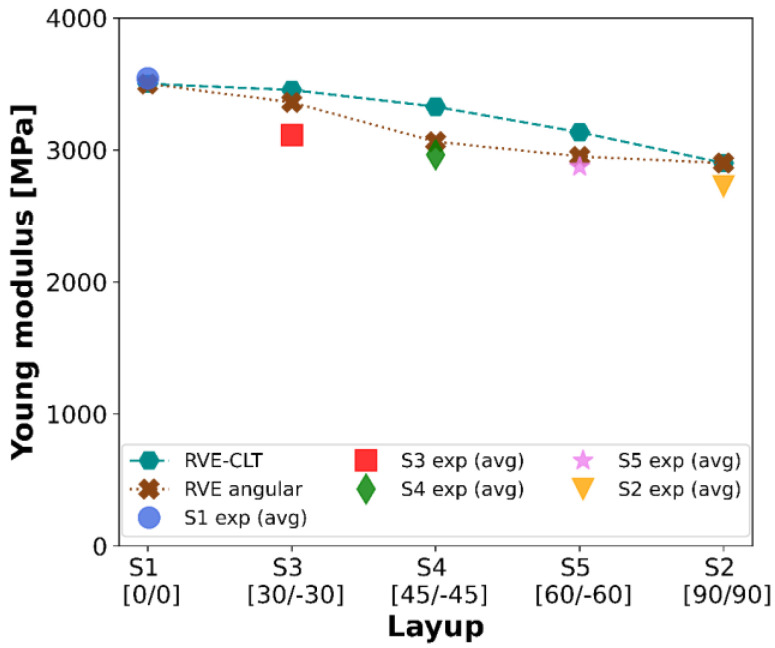
Averaged experimental results confronted with theoretical approaches.

As the final stage in this study, a graph with plots of averaged Young’s moduli with all methodologies included is shown in Figure 24.

The strength behavior along the spectrum of raster angles, namely lower raster angles, as in S4 or S5, differs less in strength than higher raster angles, as in S1 or S2. In the S3 and S4 series, experimental results correlate well with angular RVE results: 3000 MPa to 3100 MPa and 2900 MPa to 3000 MPa—in both cases, relative error maintained a lower level than 3.5%. Nevertheless, the least indefinite results between specific methods can be spotted for the S1 and S2 series, with the least approximated results for a 0° filament orientation: 3500 (experimental) and 3500 (RVE-CLT and angular RVE).

An angular RVE plot undoubtedly reflects experimental results better than an RVE-CLT plot. CLT is a methodology developed with the intention of utilizing it with composite materials. The most notable difference is layer bonding. The 3D-printed samples consist of gaps, whereas composite laminates do not. Hence, this discrepancy in results may come from the fact that FFF 3D printed material does not strictly comply with assumptions made for Classical Lamination Theory regarding composite materials.

## 5. Discussion

Before reaching numerical procedures, 3D-printed specimens were manufactured and tested using a tensile test machine. Preliminary tests proved to be necessary before conducting experiments. They helped with attaining a suitable way to produce specimens that could be used during the measurements. Applying dimensions from ASTM standards allowed for proper breaking in the case of [90,90] layup. In addition, calculations based on data obtained from measurements complied with assumptions made before attempting the study, namely that strength is conversely proportional to the raster angle.

The two numerical methodologies demonstrated in this study depict specific advantages. The RVE-CLT method requires less work in numerical software, as it contains only 6 simulations, whereas the angular RVE needed 36 × 6 = 216 simulations. On the other hand, the latter omits the issue with the shear modulus that arose in the RVE-CLT method. The Finite Element Method is based strictly on the accuracy of the discretization of a 3D model, in this case, the RVE. A higher number of elements was implemented in the second approach; nevertheless, the simplicity of the geometry of the RVE proved that even in the RVE-CLT method, with fewer elements, the influence of discretization on the final results can be omitted.

The highest yet acceptable discrepancy among values of Young’s moduli, equal to 12.29%, was demonstrated for the RVE-CLT approach. Numerical results that were more fitting to experimental procedures were achieved with the angular RVE approach, with the highest relative error just below 8%. Conducting several simulations with a parametrized angle of force applied increased the accuracy of the results. The smallest errors were noted in the S1 and S2 series, i.e., without interchanging layers, where experimental S2, compared to both numerical methods, depicted a higher error equal to 6.38%.

Data acquired from the experimental test was used as input to an algorithm based on Classical Lamination Theory. The direct approach did not provide accurate results only in the case of the shear modulus, maintaining accuracy for Young’s moduli and Poisson’s ratios. Great attention has to be paid while analyzing analytical results, as could have been seen in this case—if not for Saint-Venant’s Principle and redefining the equation for shear modulus, the results would be inaccurate, especially since the initial stress was smaller.

Regarding the incorrect breaking of the specimens, selecting a more suitable mechanical test that would still provide necessary mechanical properties, such as inter-laminar shear or three-point bending, could overcome the problem. Analytical as well as numerical methodology ought to be adjusted in this case.

One of the most crucial factors influencing overall strength in any case investigated in this study is the extent of the void inside the sample. Fibres in the laminate, in this case, filament strips, did not fully overlap, which caused the formation of gaps between them. Decreasing the gaps’ size could positively influence the material’s mechanical properties, i.e., tensile strength or Young’s modulus. Melting temperature or general printing speed during the specimens’ manufacturing could have influenced the microstructure of the samples. For instance, a lower speed could improve gap formation. Nevertheless, fully removing gaps in FFF 3D printing brings various issues to the printing process. The microstructure shows that the cross-section of a filament strip is not a regular polygon; hence, it will not fully tessellate without creating gaps. In order to build a microstructure without gaps, the strips ought to overlap significantly, which, in turn, vastly increases printing time.

When it comes to measurement reading, the tensile test machine used in this study provided data that had to be analyzed and computed to obtain mechanical properties. Even though the machine testing range was 50 kN, offering lower accuracy in the force interval under inspection, the differences in behavior between samples with different raster angles were well visible. Later, proper mathematical algorithms prepared in the programming language Python diminished the labor work connected with the determination of these properties. On the other hand, technologies such as DIC (Digital Image Correlation) can provide an entire map of the strain field within the tested structure without direct contact with the sample, compared to the extensometer used in this study. Such reading could offer better conclusions on how strains are displaced within the sample while undergoing tensile stress.

Fused Filament Fabrication is becoming increasingly popular, even among private individuals. Since most do not have an advanced understanding of micromechanics, a rule was firmly established: G-codes are keen to be created with a 45° filament orientation. If such a model undergoes any stress, it is not pure tension, as it was tested in this study; nevertheless, it still showed that [45,−45] have a specific characteristic, that is, the highest strain before breaking.

## 6. Conclusions

In this study, 3D printed structures are tested experimentally in terms of their response to tensile stress, and numerical and analytical approaches are made to predict strength behavior in a spectrum of raster angles of filament strips in the structures. Experimental measurements—the tensile test chosen for this study was conducted, attaining accurate results but with incorrect breaking. The numerical part of the study proved that direct results taken from FEM software were unreliable and had to be altered through a non-sensitive shear modulus methodology. In addition, a more profound simulation was carried out, including parametrization. Finally, the results from all procedures were compared and analyzed. The RVE-CLT approach showed a maximum discrepancy in Young’s modulus values of 12.29%. A closer match to experimental outcomes was found using the angular RVE method, with errors up to 8% as the method turned out to be non-sensitive to the determination of shear’s moduli. Also, the study has shown that material behavior greatly depends on building parameters, mainly filament orientation. Contrary to the isotropic material type of PLA, 3D-printed specimens made with it exhibited strictly orthotropic behavior.

Research conducted in this study contributes to the determination of a suitable approach to simulate stress behavior in FFF 3D printed structures numerically. Such studies are highly important, considering the development of additive manufacturing technologies and other technologies, such as SLA or SLS. Even though the main goal of this study was achieved, there is a wide range of future opportunities for this study, such as using more sophisticated material characterization techniques (e.g., scanning electron microscope, hole-drilling technique, or dynamic mechanical analysis, allowing for mesoscale modeling). Failure simulation approaches could simulate breakage. As mentioned earlier, the [45/−45] layup exhibited particularly high strain at break that could be studied more profoundly with other layups, especially those near 45° or −45°.

## Figures and Tables

**Table 1:** G-code parameters.

Parameter	Value
Nozzle temperature	215 °C
Bed temperature	60 °C
Layer height	0.2 mm
Layer width	0.4 mm (0.2 mm)
Flow multiplier	100%
General printing speed	30 mm/s
Cooling fan speed	100%

**Table 2:** Material properties of Fiberlogy PLA, according to the technical datasheet delivered by the producer [40].

Parameter	Value
Tensile strength at yield	50 MPa
Tensile strength at break	53 MPa
Young’s modulus	3500 MPa
Elongation at yield	6%
Elongation at break	-
Specific density	1.24/cm^3^

**Table 3:** Results from preliminary measurements.

Filament Orientation	# Samples Tested	Avg. Young’s Modulus [MPa]	Standard Deviation	Avg. Failure Stress [MPa]	Standard Deviation
0°	20	3500	80	50	8
90°	14	2700	130	36	8
90° × 0°	20	3100	130	44	6

**Table 4:** RVE calculation results.

$E_{x}$ [MPa]	$E_{y}$ [MPa]	$E_{z}$ [MPa]
3500	2900	3100
$G_{yz}$ [MPa]	$G_{xz}$ [MPa]	$G_{xy}$ [MPa]
456	516	460
$\upsilon_{xy}$	$\upsilon_{yz}$	$\upsilon_{zx}$
0.28	0.35	0.35

## Data Availability

The data that support the findings of this study are available from the corresponding authors upon request.
